# Alström syndrome: A novel mutation in Saudi girl with insulin-resistant diabetes: Erratum

**DOI:** 10.1097/MD.0000000000007121

**Published:** 2017-06-02

In the article, “Alström syndrome: A novel mutation in Saudi girl with insulin-resistant diabetes”,^[1]^ which appeared in Volume 96, Issue 10 of *Medicine*, Dr. Naglaa Kamal should only be affiliated with Pediatric Hepatologist Faculty of Medicine, Cairo University, Cairo, Egypt.
